# Peroxide of Hydrogen as a Therapeutical and Bleaching Agent

**Published:** 1892-02

**Authors:** P. P. Starke

**Affiliations:** Richmond, Va.


ARTICLE III.

### PEROXIDE OF HYDROGEN AS A THERAPEUTICAL AND BLEACHING AGENT.

DR. P. P. STARKE, RICHMOND, VA.

Perhaps no remedy of recent notice, has attracted so much attention, or realized so fully the expectations, as oxygen in some forms.

The most notable feature in regard to oxygen is the entire freedom with which it may be used on the tissues of the body without injurious effects, and that, while it is held in the greatest esteem by many, it is *condemned by none.* This cannot be claimed for any other remedy. Oxygen has no enemies, and if it had, being nature's own remedy, it would eventually triumph, notwithstanding man's tendency to ignore it in the treatment of disease.

For all practical purposes we are surrounded by an atmosphere of oxygen to which we are, doubtless, more indebted for the recovery from disease and the healing of wound than to the medical man with his pills and potions and multitudinous antiseptics. But experience has demonstrated that oxygen, at the moment of liberation from any of its compounds, having an unsatisfied affinity, is more active than in its free state as found in the atmosphere.

Peroxide of hydrogen is a very unstable chemical compound, from which oxygen is readily liberated by contact with substances having an affinity for it. The most probable impurities to be found in it are sulphuric and hydrochloric acids. The presence of the former in an objectionable amount may be determined by the cautious addition of baryta water, which gives a white insoluble precipitate of the sulphate of barium, if sulphuric acid is present. In like manner, by adding a solution of nitrate of silver, a white, insoluble precipitate of chloride of silver is formed if hydrochloric acid is there.

Peroxide of hydrogen, when properly applied, may be considered one of the most active, as well as harmless agents which we possess for sterilizing putrifying substances, after which it is without any further action on the tissues—in the manner differing from creosote, carbolic acid, bichloride of mercury and any other antiseptics which depend on their poisonous property and caustic, irritating effects for their antiseptic virtues, and are attended with danger in their concentrated form.

An ideal therapeutic agent in the treatment of septic conditions is one that may be used freely on the tissues, and not only arresting the fermentative process preceding organic decomposition, but, also, sterilizing and disinfecting the products already formed. There is no remedy in present use which fills this want so well as peroxide of hydrogen. While, according to Bert and Reynard, the soluble ferments, such as the saliva, gastric juices and pancreatic fluids, continue to act in solution of peroxide of hydrogen, purulent secretions are instantly dispelled in the form of gas on being brought in contact with it. Such has been my experience after experimenting with all forms of pus and septic matter I have been able to procure. Purulent secretions, as found in boils or festered wounds, when brought in contact with peroxide of hydrogen, produce a rapid reaction by disengagement of gas, and are thrown up in a white frosted form, almost identical in appearance to the white of an egg which has been thoroughly whipt. Likewise, the more slowly, the hard, clotted mucus taken from the nostril may be acted on, being first softened and discolored, and finally thrown off as a white froth. Decayed matter found in the crown cavities and pulp canals of the teeth, under its action give off rapidly bubbles of gas, leaving that portion which is not ejected in the form of gas in a softened condition, which, in most cases, detaches readily under the use of the excavator, and coming away with ease. This is a decided advantage, as decayed matters which sometimes adhere so tenaciously to the sides of the walls of a cavity may be softened to such an extent as to peel off easily under the use of the instrument.

We will often find, also, that cavities which appear on examination by the eye or mirror to be perfectly prepared, give off bubbles of gas on being treated with peroxide of hydrogen, thus showing the cavities, which have been prepared to the best of our judgment, may yet contain septic material. This is specially apt to occur in white decay, where it is impossible for the eye to distinguish between the sound and decayed structures, and where, in spite of all efforts, there is often a failure to thoroughly disinfect the parts. Here this agent cannot be too highly prized. Indeed, it would be well if we kept a constant supply in our cabinet, and used it in every time before filling—letting it remain in the cavity for a minute or so before filling, or so long as bubbles continue to rise to the surface. In this manner cavities, which apparently are perfectly prepared and ready to receive filling will, many times, be found to contain infectious matter.

In treating a diseased surface in the mouth, it is customary, before applying the therapeutical agents, to cleanse the parts of clotted mucus and adhering secretions by the use of warm water, so that the remedies used may come in contact with the tissues, for, cold water not dissolving or removing the clotted secretions of the mouth or from wounds, the remedies used do not come in free contact with the tissues unless they are of such a corrosive nature as to eat their way through. Here peroxide of hydrogen has no equal, though, on the score of economy, warm water may be found useful to remove mechanically the coarser portions of collected matters, it should be left to peroxide of hydrogen to go over the parts and remove chemically all purulent collections of minutest kind, which it does perfectly.

Prof. Gorgas, in his "Dental Medicine," describes its application as entirely painless. This will be found true, except where it is injected into the nasal passages, where it produces a sharp, stinging sensation, requiring its dilution till the pain is reduced. But, as cool water produces pain when used here, it may be attributed more to the peculiarity of the sensitive nerves of this locality than to the properties of the drug.

By its use as a spray from the atomizer, chronic or acute catarrh may be cured. For which purpose it should be diluted till the stinging sensation becomes bearable, after which it is best to use it of as great strength as possible.

It may be relied on as the most valuable antiseptic and disinfectant in use in the treatment of abscesses, access being made first to the cavity of the abscess, and the hydrogen peroxide injected, and repeated till no reaction occurs; any remedy being liable to disrepute if placed in a crown cavity and expected to cure an abscess at the extremity of the root.

The good results obtained by the use of the eucalyptus and iodoform in the treatment of abscesses and wounded surfaces is thought by some to be caused more to its therapeutical influences over the tissues than to its antiseptic virtues. And, according to Dr. Black, in April *Cosmos*, a series of experiments have shown that the bacteria found in the cavities of decayed teeth continue to develop in solutions of eucalyptus or iodoform. But, as the drugs are often combined with others, and their use preceded by the free use of water, it is hard to determine which exerts the most beneficial influence. The advocates of hydrotherapy will give the credit to water, those favorable to eucalyptus to that drug, while the general surgeon is willing to stake his reputation on the iodoform.

Peroxide of hydrogen is, probably, the only agent which has detergent, antiseptic, disinfectant and non-poisonous properties combined with a healthy therapeutical influence over the tissues. As a detergent I have used it in conditions of the mouth where the gums and mucus membrane were covered with clammy, sticky coating of mucus, which adhered very tenaciously, cleaning them off thoroughly and leaving the parts beneath clean and pure. As an antiseptic and disinfectant, to get an idea of its value it may be composed with sulphur dioxide, S O2. If a tooth, having a large cavity still containing the *debris* from the decomposed structures, be taken and filled with peroxide of hydrogen, bubbles of gas will immediately commence to ascend rapidly to the surface, showing that a septic condition is present. If this is rinsed as soon as the action commences and before it has time to complete it, after treating with sulphur dioxide, S O2, for a short time, no bubbles of gas are given off on the reapplication of hydrogen peroxide. This shows conclusively that it has antiseptic and disinfectant properties; for, as soon as the *debris* is disinfected, peroxide of hydrogen is without action on it, while before such treatment it was extremely active. In regard to its non-poisonous properties, I have, myself, swallowed it freely without the least injurious results that I could determine.

In regard to its therapeutical influences over the tissues, we may quote the conclusions of Bert & Reynard. They say: "that peroxide of hydrogen, even when very dilute, arrests fermentations due to the development of living organisms, and the putrefaction of all substances which do not decompose it; that containing, according to circumstances, from two to six times its volume of oxygen, it is capable of advantageously replacing alcohol and carbolic acid; that it can be employed externally for dressing wounds and ulcerations of all natures, in injections, and in vaporizations, and internally; that the results obtained in the largest operations, up to the present. are in the highest degree satisfactory; that not only fresh wounds, but old ones, proceed rapidly to cicitrization, and reunion by first intention appears to be encouraged by its use as a dressing; that the general, as well as the local, state appears to be favorably influenced; that the advantages over carbolized water are its not having any poisonous effect nor unpleasant odor, while its application is entirely painless. It is an effective application in a large class of diseases in which mucous tissue is chiefly affected, and for cleaning purposes, is considered to be unequalled. "It is employed as an internal remedy in low forms of fever, chronic and sub-acute rheumatism, whooping cough, chronic bronchitis, dyspepsia, as it improves digestion, diabetes, etc." "It acts very promptly in feeble, flabby or ill-conditioned ulcers, chancre, and diphtheritic sores, ozoena, wounds, both flesh and putrid, etc."

Peroxide of hydrogen is also useful as a bleaching agent for discolored teeth, and has the advantage of being entirely harmless and without chemical action on the structures of the tooth; is not irritating to the tissues of the mouth; does not itself produce another form of discoloration; requires no special instrument for the introduction of fillings after its use, nor the removal of adjacent fillings to prevent chemical action on their metals with resulting stains.

When practicable, it should be used to the exclusion of all other agents in those cases requiring an oxidizer to remove discoloration, it being objectionable to use other oxidizing agents which weaken tooth structure, or within themselves are liable to produce another form of discoloration, which is, at times, almost impossible to be gotten rid of. It is not claimed for peroxide of hydrogen that it will bleach all forms of discoloration; no one agent will do this; but it should be used in all cases except where, from the nature of the discoloration, a special bleaching is required to chemically combat the peculiar stain deposited in the tubuli, as the cyanide of pottasium to remove silver stains, oxalic acid to remove discolorations from manganese dioxide, and similar cases, in which only a chemical compound is effective. The bleaching action of hydrogen peroxide appears to be exerted on the decomposed animal matter or mucous stains. It acts on the block sulphid of lead ($Pb S$) and converts it into the white sulphate of that metal ($Pb SO_4$). In bleaching, after taking away with the excavator as much of the decomposed and discolored material as is desirable, the cavity of the tooth should be filled with a solution as concentrated as possible, and refilled till no bubbles of gas are given off on applying it. Or it may be applied on cotton which is saturated with it, and reapplied as often as necessary. The weaker the solution used the more stable it will be found, but requiring a greater length of time in bleaching in proportion to its dilution.

The presence of acids retard, and alkalies hasten, its action, consequently our time may be economized by the addition of such a quantity of a solution of lime water or bicarbonate of soda as will render it alkaline in reaction.

Observation as to the length of time in bleaching are liable to differ, if we do not take into consideration the percentage of the solution used. For by calculation, we readily see that a concentrated solution of 106 per cent. would bleach in one hour to an extent requiring twenty-five by a 4 per cent. solution. But it does not follow that because we buy a solution containing a large per centage of oxygen that it will retain such a strength, as left in bottles unstopt, or at a temperature higher than 70 F., it losses its oxygen.

The following table will show the amount of oxygen found in solutions of peroxide of hydrogen of varying percentage:

| | | | | | | | | |
|---|---|---|---|---|---|---|---|---|
| 100 | gr. of a | 100 | per cent. solution of | hydrogen | peroxide | contains | 47.6 | gr. O. |
| 100 | " | 50 | " | " | " | | 23.8 | " |
| 100 | " | 25 | " | " | " | | 11.9 | " |
| 100 | " | 12 | " | " | " | | 5.712 | " |
| 100 | " | 4 | " | " | " | | 1.904 | " |
| 100 | " | 2 | " | " | " | | .953 | " |

The above shows that if a 2 or 4 per cent. solution is used, the action must necessarily be extremely slow as compared with a concentrated solution.

The danger of using permanganate of potassium as a bleaching agent is referred to by Dr. E. C. Kirk, of Philadelphia, in the June number of the *Items of Interest*, as follows:

"In the case of potassium permanganate, there results, among other manganese dioxide, MN O2, a dark brown solid, which in itself produces a discoloration that must be gotten rid of afterward by strong solution in oxalic acid, with which it forms an almost colorless and soluable compound. I have used this substance for bleaching teeth, but care must be taken not to use it in a very concentrated solution, otherwise the final treatment with oxalic acid may fail to remove the discoloration from the manganese dioxide which has been precipitated in the tubuli, and leave the tooth in a worse condition than the first. Used with care, in dilute solutions of a claret color, and almost immediately followed by a strong oxalic acid or binoxalate of potassium solution, I have obtained good results where the tooth structure to be bleached was not very thick or dense."

The use of chlorin in any form, while the most universal, is, without doubt, the most injurious agent on tooth structure which could possibly be selected from our present list of bleaching agents.

The following precautions, according to Professor Truman, of Philadelphia, should be observed:

"The treatment of the tooth previous to bleaching is the same for all methods. The upper third of the pulp canal should be solidly filled with gutta-percha. Gold has been recommended, but should not be used in any tooth bleached by chlorine, as it is attacked by the latter, and the auric chloride formed decomposes in the presence of organic matter by the action of light and oxygen, and results in a permanent purple stain which cannot be gotten rid of. For this reason a tooth to be bleached should have gold fillings removed if they are in position.

"The cavity should in all cases be washed out with ammonia or borax to remove fatty matter, and no substance which has the power to coagulate the albumen should be used, as such prevents the ingress of chlorine to the tubuli. For the final washing, distilled water should be used, as river water, ordinarily, contains sufficient iron to stain the tooth in combination with the chlorin as ferric chlorid.

"Lastly, after the bleaching is completed, the cavity and pulp canal should be filled with oxychloride of zinc, which should be inserted with instruments of bone, hard rubber or wood. It should be carefully borne in mind that no metallic instrument should come in contact with the tooth after the chlorin has been applied."

The foregoing precautions so limit the actions of the practitioner of dentistry as to render the use of chlorin in bleaching teeth as an act which should be resorted to only in desperate cases, for we note that gold fillings in position should be removed to avoid discoloration from auric chlorid; that only oxychlorid of zinc or gutta-percha may be used as filling materials, and that only instruments of bone, rubber or wood are admissible in applying the filling material, as metallic instruments should not be brought in contact with the bleached surface, for fear of producing a different form of discoloration.

But such are not all the objections to the use of chlorin for this purpose, that of injuring the tooth structure and irritation to the throat of the patient being important considerations. The hydrochloric acid formed during the bleaching process by chlorin is, probably, the most active destroyer of tooth structure that can be selected, a solution of this acid completely destroying a tooth in from 10 to 20 hours, though a concentrated solution of sulphuric acid requires about the same number of days to accomplish the same end. Therefore, the conclusion we arrive at is, that chlorin is the most injurious agent which can be used for bleaching purposes; sulphur dioxide, $SO_2$ which is a valuable disinfectant, less injurious, and peroxide of hydrogen least injurious, or, rather, not injurious, but decidedly beneficial, whether it is in the cavity of a tooth or the throat of a patient.

The demand for peroxide of hydrogen being small, it is difficult to produce it in just any percentage of strength desired and at the low price at which it might be obtained if it had a large sale, as the materials from which it is manufactured are cheap.

To obtain the best results from peroxide of hydrogen, the following points should be borne in mind:

1. That it should be kept in glass stoppered bottles at a temperature not higher than 70° F., though when diluted it may be kept at a higher temperature without being decomposed.

2. The presence of acids retard, and alkalies hasten, its action.

3. That its action is quickened in proportion to its concentration, and that if used in very small strength for bleaching, it may require a length of time greater than the dentist or patient is willing to give.

4. The most common impurities being sulphuric and hydrochloric acids, the sulphuric acid may be detected by the addition of a small quantity of baryta water, and the hydrochloric acid by a solution of nitrate of silver, when, in either case, a white precipitate will be formed if the acid indicated be present.

5. When used to clear the nasal passages, the stinging sensation may be reduced by dilution, and heated to the temperature of the body.

6. That when used in abscesses or other diseased conditions, it should be carried to the parts affected, as, unlike carbolic acid or creosote, it possesses no curative powers when applied to remote parts as a counter-irritant.

7. Re-apply as long as bubbles of gas are given off.

By carefully following out each of the preceding rules, peroxide of hydrogen is an indispensable article in the dental office.—*Southern Dental Journal.*

